# Evaluating health facility access using Bayesian spatial models and location analysis methods

**DOI:** 10.1371/journal.pone.0218310

**Published:** 2019-08-07

**Authors:** Nicholas J. Tierney, Antonietta Mira, H. Jost Reinhold, Giuseppe Arbia, Samuel Clifford, Angelo Auricchio, Tiziano Moccetti, Stefano Peluso, Kerrie L. Mengersen

**Affiliations:** 1 Queensland University of Technology, Department of Statistical Science, Mathematical Sciences, Science & Engineering Faculty, Brisbane, Queensland, Australia; 2 ARC Centre of Excellence for Mathematical and Statistical Frontiers (ACEMS), Brisbane, Queensland, Australia; 3 Department of Econometrics and Business Statistics, Monash University, Melbourne, Victoria, Australia; 4 Data Science Center, Institute of Computational Science, Università della Svizzera italiana, Lugano, Switzerland; 5 Department of Science and High Technology, Università dell’Insubria, Como, Italy; 6 Department of Statistical Sciences, Università Cattolica del Sacro Cuore, Milan, Italy; 7 Department of Infectious Disease Epidemiology, London School of Hygiene & Tropical Medicine, London, United Kingdom; 8 Centre for Mathematical Modelling of Infectious Diseases, London School of Hygiene & Tropical Medicine, London, United Kingdom; 9 Fondazione Ticino Cuore, Lugano, Switzerland; 10 Division of Cardiology, Fondazione Cardiocentro Ticino, Lugano, Switzerland; 11 Center for Computational Medicine in Cardiology, Università della Svizzera Italiana, Lugano, Switzerland; The University of the South Pacific, FIJI

## Abstract

**Background:**

Floating catchment methods have recently been applied to identify priority regions for Automated External Defibrillator (AED) deployment, to aid in improving Out of Hospital Cardiac Arrest (OHCA) survival. This approach models access as a supply-to-demand ratio for each area, targeting areas with high demand and low supply for AED placement. These methods incorporate spatial covariates on OHCA occurrence, but do not provide precise AED locations, which are critical to the initial intent of such location analysis research. Exact AED locations can be determined using optimisation methods, but they do not incorporate known spatial risk factors for OHCA, such as income and demographics. Combining these two approaches would evaluate AED placement impact, describe drivers of OHCA occurrence, and identify areas that may not be appropriately covered by AED placement strategies. There are two aims in this paper. First, to develop geospatial models of OHCA that account for and display uncertainty. Second, to evaluate the AED placement methods using geospatial models of accessibility. We first identify communities with the greatest gap between demand and supply for allocating AEDs. We then use this information to evaluate models for precise AED location deployment.

**Methods:**

Case study data set consisted of 2802 OHCA events and 719 AEDs. Spatial OHCA occurrence was described using a geospatial model, with possible spatial correlation accommodated by introducing a conditional autoregressive (CAR) prior on the municipality-level spatial random effect. This model was fit with Integrated Nested Laplacian Approximation (INLA), using covariates for population density, proportion male, proportion over 65 years, financial strength, and the proportion of land used for transport, commercial, buildings, recreation, and urban areas. Optimisation methods for AED locations were applied to find the top 100 AED placement locations. AED access was calculated for current access and 100 AED placements. Priority rankings were then given for each area based on their access score and predicted number of OHCA events.

**Results:**

Of the 2802 OHCA events, 64.28% occurred in rural areas, and 35.72% in urban areas. Additionally, over 70% of individuals were aged over 65. Supply of AEDs was less than demand in most areas. Priority regions for AED placement were identified, and access scores were evaluated for AED placement methodology by ranking the access scores and the predicted OHCA count. AED placement methodology placed AEDs in areas with the highest priority, but placed more AEDs in areas with more predicted OHCA events in each grid cell.

**Conclusion:**

The methods in this paper incorporate OHCA spatial risk factors and OHCA coverage to identify spatial regions most in need of resources. These methods can be used to help understand how AED allocation methods affect OHCA accessibility, which is of significant practical value for communities when deciding AED placements.

## Introduction

To best serve the public, hospitals, medical centres, and emergency services should be in locations where they can serve the most people in need. Recent research has evaluated various techniques for evaluating or identifying locations of External Defibrillators (AEDs) [1, 2]. This is an idea upon which this paper builds: exploring how to evaluate, locate, and improve AED locations.

AEDs are a portable device that can be used by a layperson to provide advanced life support for out of hospital cardiac arrests (OHCAs) by bringing the device to the victim. OHCAs are a major public health problem affecting about 1 citizen per 1000 inhabitants in developed countries [3–5]. OHCA survival decreases up to 10% for each minute of delay between collapse and treatment, but survival can be improved through delivery of cardiopulmonary resuscitation (CPR) from bystanders, and early defibrillation [6] through devices such as AEDs. Bystander response to OHCA events improve OHCA survival by performing CPR and providing advanced life support using AEDs [3, 4]. The impact of bystander AED use is increasing with technology such as smartphone applications being used to assist in responding to and treating OHCA events, improving survival [7].

AEDs need to be close to OHCA events so they can be used quickly in response to an OHCA event—ideally within 100m or a 2 minute walk to provide adequate coverage [2, 8, 9]. AEDs are currently placed following methodology prescribed by the American Heart Association (AHA) and European Resuscitation Council (ERC), with AEDs being placed where OHCA events occur every 2 and 5 years, respectively. However, it is prohibitively expensive to place AEDs where all OHCA events occur. Alternative methods for AED placement need to be considered.

Population-based strategies place AEDs in locations with high observed OHCA occurrence [10], but these may not generalise well to other areas. For example, golf courses had high OHCA incidence in one region, but not another [2, 10, 11]. This motivates the need for more sophisticated AED placement strategies.

AED access can be improved by modelling access as a relationship between supply (AEDs locations), and demand, (OHCA events). This can be achieved using a method known as the two-step floating catchment area (henceforth 2SFCA), a special case of the gravity-based model, which has been used to measure healthcare access [12]. As the name suggests, the 2SFCA involves two steps. The first step calculates the supply-to-demand ratio for each supply point, which measures how well the supply meets the demand. The second step calculates the access for the spatial catchment.

A modification of the floating catchment approach has been applied to identify priority regions for AED placement based on supply of AEDs and demand of OHCA events [1]. Here, the authors incorporated exponential weights to account for the step-wise decay of distance access in a given catchment, to give more weight to OHCA events that are closer to AEDs, rather than equally weighting them. Demand was measured by incorporating risk factors for OHCA occurrence, such as age, gender, income, and land use information. A Bayesian geospatial model was used to account for these factors, and estimate the count of OHCA events, which were then used to compute access in a geospatial region. Following this, the authors identified priority areas for AED placement based on the supply-to-demand ratio. Their approach is incomplete, however, as it does not identify exact AED locations, or provide methods for precise placement. This means AEDs could be poorly placed within a region identified as priority. There is a missed opportunity to compare and combine information from priority regions with exact ideal AED locations.

Mathematical optimisation strategies have improved AED access [2, 8, 9]. This approach identifies precise AED locations that optimally cover as many OHCA events in a set distance (e.g., 100m) as possible. These models are based on the Maximal Covering Location Problem, originally described by Church and Velle [13]. The approach has been applied to AED placements by Chan et al [8], where they were shown to be more effective than population-based approaches. This approach is referred to as a fixed location method, as AED locations are fixed and cannot be relocated. Recent research [14] has further validated fixed-location optimisation strategies, demonstrating that it is effective in rural and urban areas. Additionally, they demonstrated the potential efficacy of a cost effective relocation approach, where existing AEDs are relocated to cover more OHCA events, covering up to 50% of OHCA events.

These two approaches for AED placement work at different scales and use differently structured information. The first incorporates spatial covariates related to OHCA occurrence, and OHCA and AED locations to identify priority regions for AED placement, but does not identify precise AED locations. The second approach identifies precise AED locations based on previous OHCA events, but does not consider other known risk factors for OHCA, such as age, population density, and gender [1, 15, 16] into the AED placements. It is currently unknown whether these optimisation approaches are allocating AEDs to regions needing access, only that they improve coverage. The AED placements also do not take into account other more global spatial predictors of OHCA occurrence. These two approaches could be used together to jointly evaluate the impact of AED placements and drivers of OHCA occurrence. This provides a more comprehensive approach and identifies precise AED placements, priority areas for AED placements, and describes drivers of OHCA events.

This paper has two aims. The first is to develop geospatial models of OHCA, accounting for and displaying uncertainty. The second is to evaluate the AED placement methods using geospatial models of accessibility. To achieve our aims we identify communities with the greatest gap between demand and supply for allocating AEDs, and then use this approach to evaluate models for precise AED location deployment.

This paper proceeds as follows, we first describe the case study data and data processing. Then, we explain how spatial effects are measured, how access is measured, and how we measure access and priority ranking. We then describe the optimal allocation of AEDs. Results are then presented, and discussed, along with ideas for future research and final conclusions.

## Materials and methods

### Ethics

Data are collected and stored following Good Clinical Practice Guidelines and the relevant legislation governing the use of patient data. The investigation complied with the Declaration of Helsinki”s principles for physicians engaged in biomedical research involving human subjects. The Queensland University of Technology Human Research Ethics Committee assessed this research as meeting the conditions for exemption from HREC review and approval in accordance with section 5.1.22 of the Australian National Statement on Ethical Conduct in Human Research.

### Data

Data in this study can be broken up into two different categories: (1) OHCA and AED related data, (2) and spatial and statistical data.

#### Data: OHCA and AED related data

OHCA was data obtained from a cardiac arrest registry based in Ticino, Switzerland, which has a population of 346,539 (as of 2013) and covers 2812 km^2^ [17]. The data contains OHCA events from January 1st 2005 to December 31st 2015 for individuals older than 1 year. OHCA events in this registry are defined as events that occur outside of a hospital, where there is cessation of cardiac mechanical activity, and is confirmed by the absence of signs of circulation. OHCA events are geolocated as GPS latitude and longitude co-ordinates. A small number of cases matched to written addresses, which places events in the centre of a street or suburb. Data for AEDs contain GPS co-ordinates and availability: either public and available 24 hours a day 7 days a week, or time limited where AEDs are in a non-public structure. Locations for potential AEDs included GPS co-ordinates for every building in Ticino, approximately 118,000 locations. These data were provided by the Federal Statistical Office, Federal Register of Buildings and Dwellings.

#### Data: Spatial and statistical data

Shapefiles of the spatial polygons related to the 115 municipalities were made available from the Ufficio del catasto e dei riordini fondiari of Canton Ticino. General information on each municipality, including the financial strength of each area, was provided by USTAT, the Statistical Office of Canton Ticino. Financial strength was computed as a weighted average of indicators related to revenue, taxation, and the resident population. For complete details on the calculation of financial strength see Appendix A in S1 File. The land use, specifically, the number of hectares used for transport, industry and commercial use, buildings, recreation, and special urban use, was also available for each of these municipalities, and provided by the Federal Office of Statistics.

### Overview of methods

A brief overview of each step of the analysis is now provided. First, A grid is overlaid over the spatial area, the number of OHCA events in each grid cell are calculated, and each grid cell is assigned covariate information from the municipality it falls into. Next, a Bayesian geospatial model is fit to obtain an estimate of the expected number of OHCA events in a given grid cell. Following this a spatial access model is fit at each grid cell, providing each grid with an access score. The fixed location method is then used with the observed OHCA events to identify 100 optimal AED locations. Priority rankings are then created. Each of these steps is now explained in more detail.

### Data pre-processing

**Fig 1:**
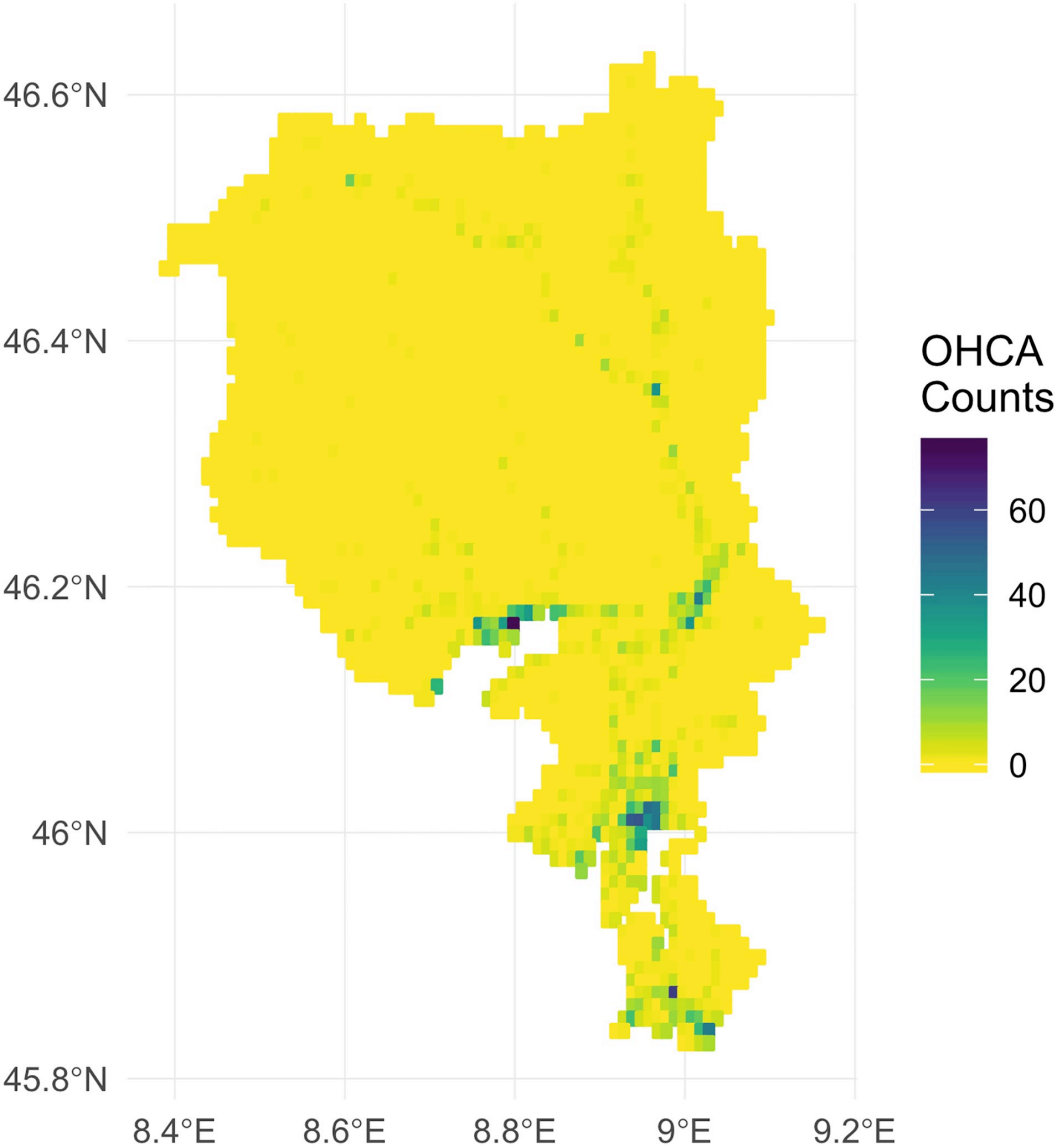
Map of the OHCA events, where the higher the number of OHCA events, the darker the bin colour.

One problem that must be tackled is that the data used in analyses are collected at different spatial scales. Specifically, OHCA events, AED locations, and building locations have GPS co-ordinates, and thus observed at the point level, while census information of the other variables are obtained at the spatial scale of municipalities. However, the municipality areas are too large for the purposes of the floating catchment analysis, so in the absence of other readily available information, a grid was generated to provide smaller resolution for analysis. This grid was defined with dimensions 0.01 degrees latitude and longitude, resulting in 3205 grid cells 1100m by 1100m (see Fig 1). The number of OHCA events that fall within each grid cell were then calculated. Covariate information for each grid cell was calculated from the municipality in which the grid cell centroid fell, and was divided by the number of cells falling within the borders of each municipality. For example, if ten grid cells fell within a municipality, the covariate values for each grid were given by dividing the municipality covariate value by ten. Fig 1 shows the grid created, and a map showing the number of OHCA events that lie within each spatial area.

### Modelling spatial effects

**Fig 2:**
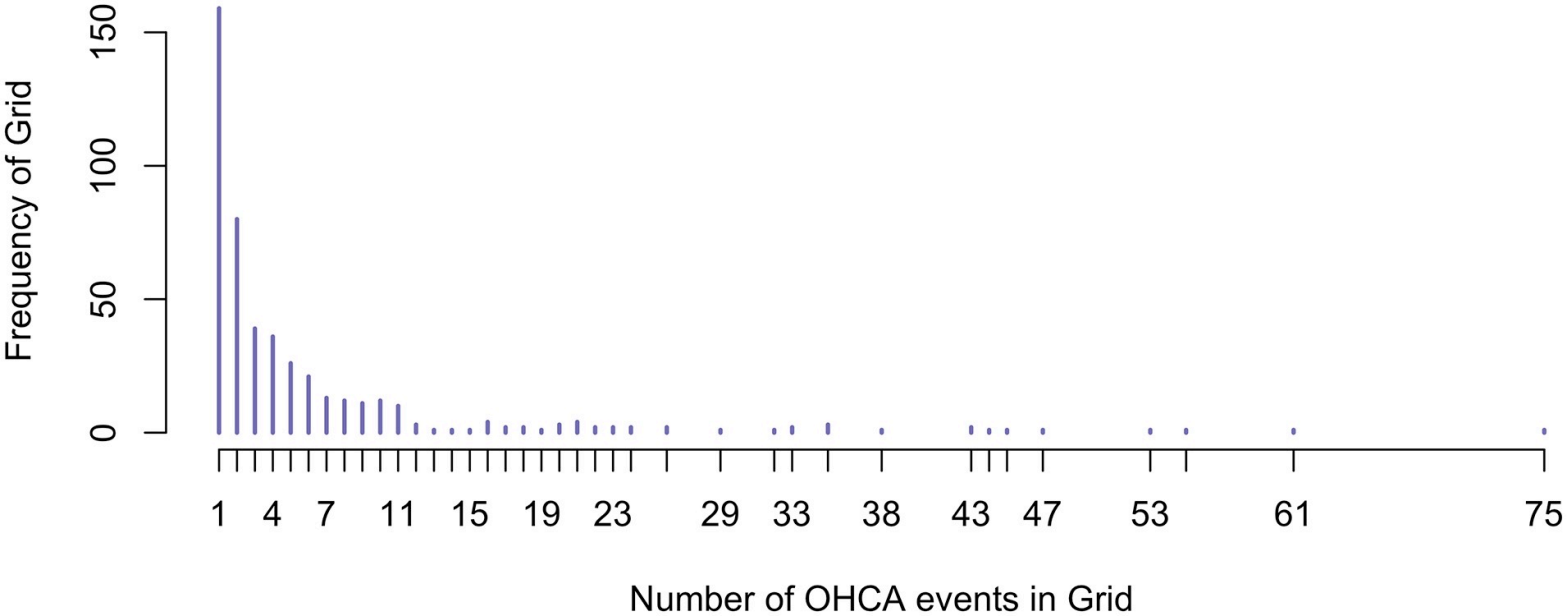
Distribution of non-zero OHCA counts in each grid cell from 2005 to 2015.

Bayesian models provide simple and effective ways of analysing small effects, and allow inferences using probabilities of potential events and outcomes. A model is fit to estimate the number of OHCAs in a given grid cell. The following covariates have been shown to be related to OHCA incidence [1, 15, 16], and so are included in the model: proportion of men, proportion of people aged over 65, financial strength of an area, and proportion of area used for transport, industry and commercial uses, buildings, recreation, and special urban use. As 85% of the spatial grid cells have zero OHCA events and many low counts (Fig 2), a Zero-Inflated Poisson (ZIP) model is used to account for these additional zeros. A ZIP model allows the number of events *y*_*ki*_ in the *i*^*th*^ grid cell in the *k*^*th*^ municipality to be zero with some probability *p*, or else to follow a Poisson distribution (which can also have zeros) with probability 1 − *p*:

$$y_{ki}∼\{\begin{array}{cc} 0, & \text{with}\;\text{probability}\;p\\ \text{Poisson}\;(\lambda_{ki}), & \text{with}\;\text{probability}\;1-p \end{array}$$ (1)

The probability *p* is expressed as:

$$p=\frac{exp(\theta )}{1+exp(\theta )}$$ (2)

and a prior is set on *θ*. In this case study *θ* was set to be θ∼N(0.000001,2). Hence a priori, *p* has a mean of 0.5, and 2.5^th^ and 97.5^th^ percentiles of 0.05 and 0.95, respectively. Other specifications of *θ* were considered (for example, θ∼N(0.001,2), and θ∼N(0.1,1)), but these had very little impact on the parameter estimates.

The parameter λ_*ki*_ was modelled as a function of the risk factors ***x*** = (*x*_1_, …, *x*_*m*_) as in Eq 3. In this model, *u*_*i*_ is a spatially structured random effect with an intrinsic conditional autoregressive specification [18, 19], and *v*_*i*_ is a spatially unstructured random effect, (see Eqs 4 and 5, respectively).

$$log\left(\lambda_{ki}\right)=\beta_{0}+\sum_{m=1}^{M}\beta_{m}x_{mi}+u_{i}+v_{i}$$ (3)

$$u_{i}|u_{j},\tau_{u}∼N(\frac{1}{n_{i}}\sum_{i∼j}u_{j},\frac{1}{\tau_{u}n_{i}}),\;i\neq j$$ (4)

$$v_{i}=N\left(0,\sigma_{v}\right)$$ (5)

Here *τ*_*u*_ is a precision parameter; *n*_*i*_ is the number of neighbours of the *i*^*th*^ grid cell, and the subscript *i* ∼ *j* refer to a cell *i* of the grid cell and to its neighbour *j* respectively. An alternative formulation that accounts for age and gender as an offset was also considered and is described in Appendix B in S1 File.

### Measuring accessibility and priority ranking

An altered 2SFCA method is applied, which uses an exponential decay from the AED to OHCA distance, as described in [1]. Our adoption of a exponential decay function extends the 2SFCA method of [1] to rank geographical regions according to their accessibility. We believe that the continuous decreasing behaviour of the demand weight is a better approximation of the reality, relative to a step-wise decreasing function. Also, the chosen decay can be interpreted in terms of demand or in terms of procedure effectiveness: after the threshold of 100m, we consider, based on literature considerations, the AED not effective to cover an OHCA that will likely divert on a different AED (therefore no demand for the first AED).

This process has two steps, and follows the notation where *i* is a grid cell, *j* is an AED point location, and *k* is a municipality. The first step calculates the supply to demand ratio, *R*_*j*_, for each AED location. The supply of *S*_*j*_ is defined as 1 as there is 1 AED, and demand is the weighted sum of the demand scores of an OHCA event from spatial area *d*_*kj*_ within distance *d*_0_ (100m) of AED *j*.

The choice of 100m is not arbitrary and is based on current literature, [2, 8, 9], and dictated by resuscitation guidelines [20]. To elaborate, without cardiopulmonary resuscitation and AED use, the probability of surviving an OHCA is reduced by 10-15% every minute of cardiac arrest. A health untrained person can run at 2 metres per second, so a distance of 500m will be covered in about 250 seconds or about 4 minutes. This means if an AED is 500 metres away, 4 minutes equals a probability to survive of slightly more than 50%, which is very undesirable. This is a further medical justification to optimize AED distribution to reduce access time to 1 to 2 minutes or up to a distance of 100 metres. Thus, the paper focuses only on bystander response of 100m. We report the results based on a different distance of 250m in Appendices F and G in S1 File, but this caused a mismatch between AED placements and OHCA predictions. Following this, we decide to focus on bystander response up to 100m.

The ratio of supply to demand is then:

$$R_{j}=\frac{S_{j}}{\sum_{k\in \{d_{kj}\leq d_{0}\}}D_{ki}G\left(d_{kj},d_{0}\right)}$$ (6)

Here, the estimated demand score, *D*_*ki*_ for the OHCA in grid cell *i* of municipality *k*, is weighted by a exponential function with a smooth decay, set to be equal to zero at *d*_0_:

$$G\left(d_{kj},d_{0}\right)=\{\begin{array}{cc} \frac{e^{-\frac{1}{2}\times {\left(\frac{d_{kj}}{d_{0}}\right)}^{2}}-e^{-\frac{1}{2}}}{1-e^{-\frac{1}{2}}}, & d_{kj}\leq d_{0}\\ 0 & d_{kj}>d_{0} \end{array}$$ (7)

An alternative, stepwise formulation was considered, but the results were effectively identical to the exponential decay (see Appendices E, F, and G in S1 File).

In the second step, access, *A*_*i*_, aggregates this information, such that for each spatial area *i* is calculated by summing up the weighted supply-to-demand ratios for each AED that fall within spatial area *i*:

$$A_{i}=\sum_{j=1}^{J}R_{j}$$ (8)

Priority ranks are created by considering regions with predicted OHCA counts greater than 1 in a grid cell, then ordering by the lowest access score and highest predicted OHCA counts. To find the areas most in need of resources, we consider the top 20 priority areas and then rank them according to those that had the most AEDs placed.

### The optimal allocation method for AEDs

AED locations are identified using the fixed location method optimisation and the observed OHCA events. This identifies a set of AED locations that maximize the number of OHCA events covered within a set distance of an AED. Possible AED locations are given by the database of buildings in Ticino. A number of new AEDs can be specified, for example, the top 100 AED locations covering the most historical OHCA events.

More formally, the variables *x*_*j*_, and *y*_*i*_ are binary, where *x*_*j*_ is equal to 1 when OHCA *j* is covered, and 0 otherwise for *j* = 1, …, *J* OHCA events. Similarly, *y*_*i*_ is 1 if an AED is placed in location *i*, and 0 otherwise, for possible AED locations *i* = 1, …, *I*. The matrix *A* has *J* rows of OHCA incidents, and *I* columns of potential AED locations. Here, *a*_*ji*_ is binary, and indicates whether OHCA *j* is covered (within 100m) by location *i*.

$$A=\left[a_{j,i}\right]=(\begin{array}{cccc} 0_{1,1} & 1_{1,2} & \cdots & 0_{1,I}\\ 1_{2,1} & 0_{2,2} & \cdots & 1_{2,I}\\ \vdots & \vdots & \ddots & \vdots \\ 0_{J,1} & 1_{J,2} & \cdots & 1_{J,I} \end{array})$$ (9)

This optimisation model maximizes the total number of OHCAs covered by the configuration of the AEDs *y*_*i*_,

$$\text{max}\sum_{j=1}^{J}x_{j}$$ (10)

subject to the constraints: only N locations for AED placement are selected,

$$\sum_{i=1}^{I}y_{i}=N$$ (11)

and that when at least one AED *i* covers an OHCA *j*, and the corresponding AED *y*_*i*_ is selected, then OHCA *x*_*j*_ is covered.

$$x_{j}\leq \sum_{i=1}^{I}a_{ij}y_{i}\forall_{j=1\ldots J}$$ (12)

OHCAs that are already covered by current AED placements are removed from the analysis, to improve only coverage of uncovered OHCAs. The fixed location model is performed for N = 100, to find the top 100 locations for AEDs.

#### Computation

The R statistical and programming environment [21] was used for all analysis and visualisation, along with the integrated design environment, RStudio [22]. Reproducibility was ensured using the rmarkdown [23] and knitr [24] packages. The R package, maxcovr [25] was used to determine optimal AED locations, using lpSolve internally to as the linear programming solver for the fixed location method [26]. Data read in was performed using readr and readxl [27, 28], and data manipulation and results extraction used R packages dplyr, tidyr, purrr, and kableExtra [29–32]. Spatial and statistical analysis was performed using the packages simple features, sp, spdep and raster [33–36]. The ZIP model was fit using Integrated Nested Laplace Approximation (INLA), using option “zeroinflatedpoisson1” [37].

## Results

Among the 2802 OHCA cases in this study, over 70% of OHCA events occurred in those aged over 65 (Table 1). Additionally, the majority of events occurred in urban areas (Table 2).

**Table 1:** Age distribution of OHCA patients.

Age (Years)	N	Percent
0-15	3	0.11
15-64	742	26.48
65-105	2044	72.95
(Missing)	13	0.46

**Table 2:** Top 10 Municipalities by OHCA count, also indicating whether they are urban or rural areas.

Municipality	Urban / Rural	Count	Percent
69	Urban	489	17.45
66	Urban	130	4.64
78	Urban	127	4.53
13	Urban	126	4.50
36	Urban	70	2.50
51	Rural	67	2.39
7	Rural	64	2.28
49	Rural	60	2.14
82	Rural	60	2.14
14	Urban	59	2.11

**Fig 3:**
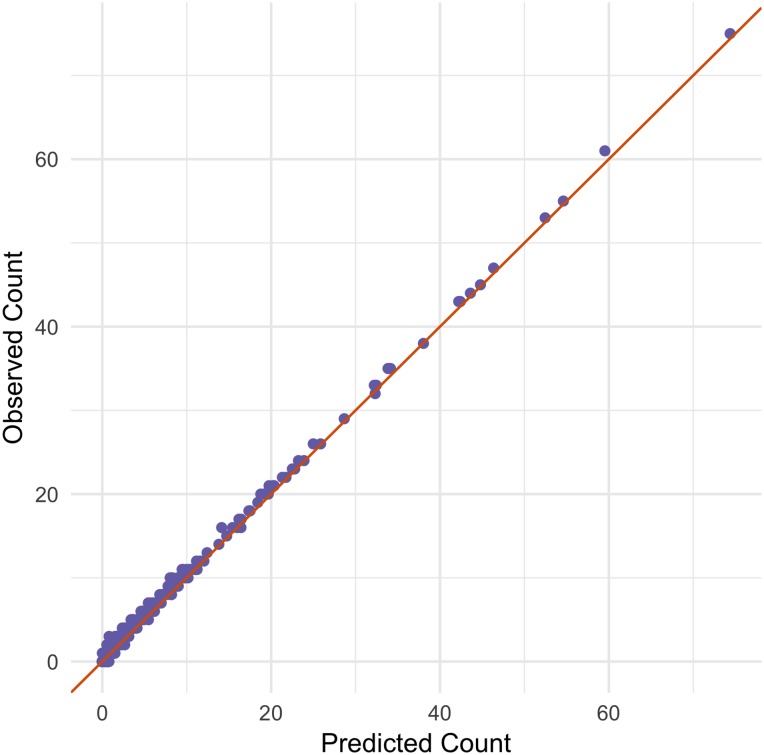
Observed counts of OHCA events in each grid cell compared to the posterior mean counts in a grid cell, with a line of perfect fit.

**Fig 4:**
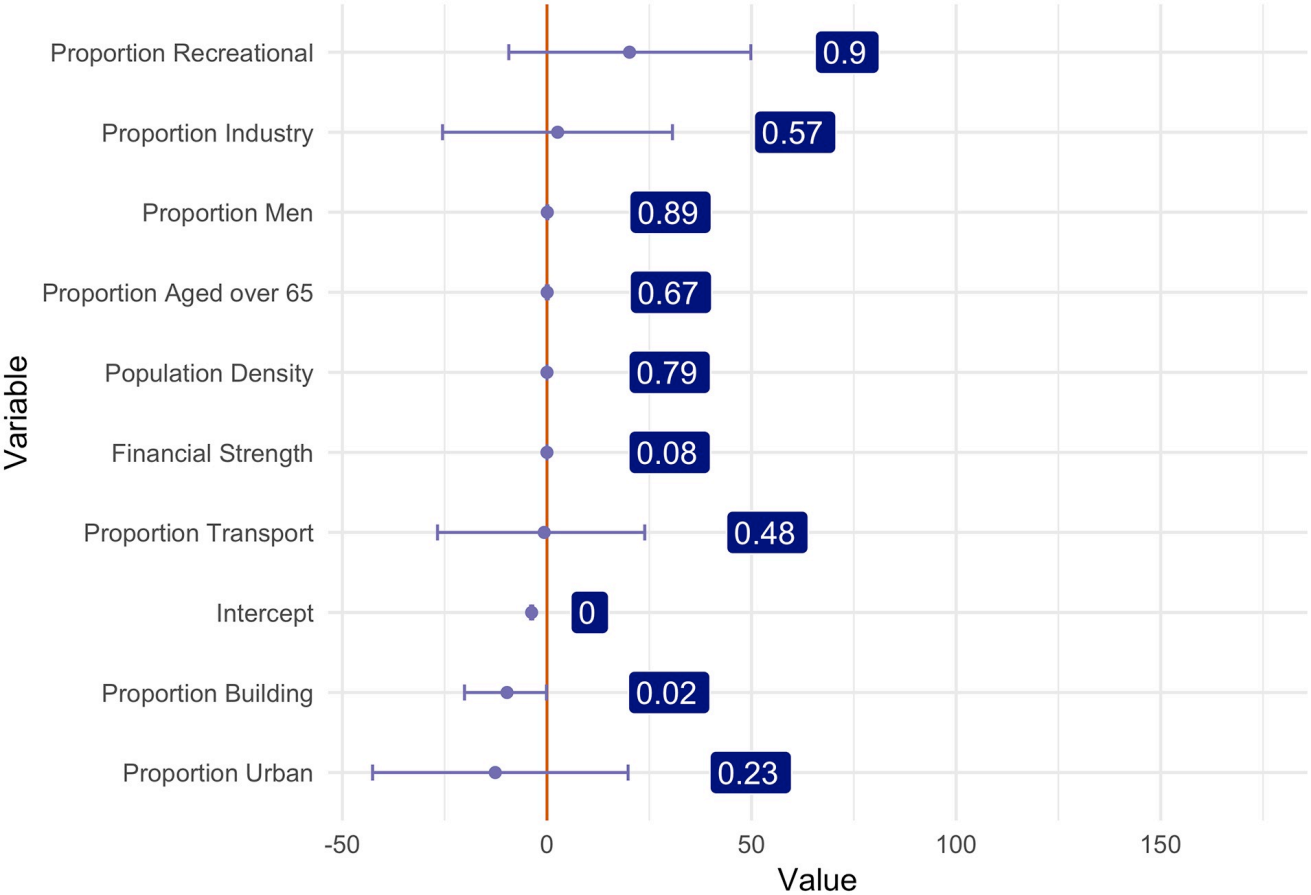
Posterior mean, 95% credible intervals, and probability of effect being > 0 for the model covariates.

The model performs well at predicting expected counts in a region (Fig 3). Fig 4 uses samples from the posterior density of each covariate, and shows the 95% credible intervals, and the probability of the covariate being greater than zero in a grid cell. Note here that recreational and industrial areas, as well as the proportion of men, being older than 65, and the population density increase OHCA occurrence. Additionally, the proportion of buildings, urban areas, and financial strengths are associated with lower counts of OHCA events.

**Fig 5:**
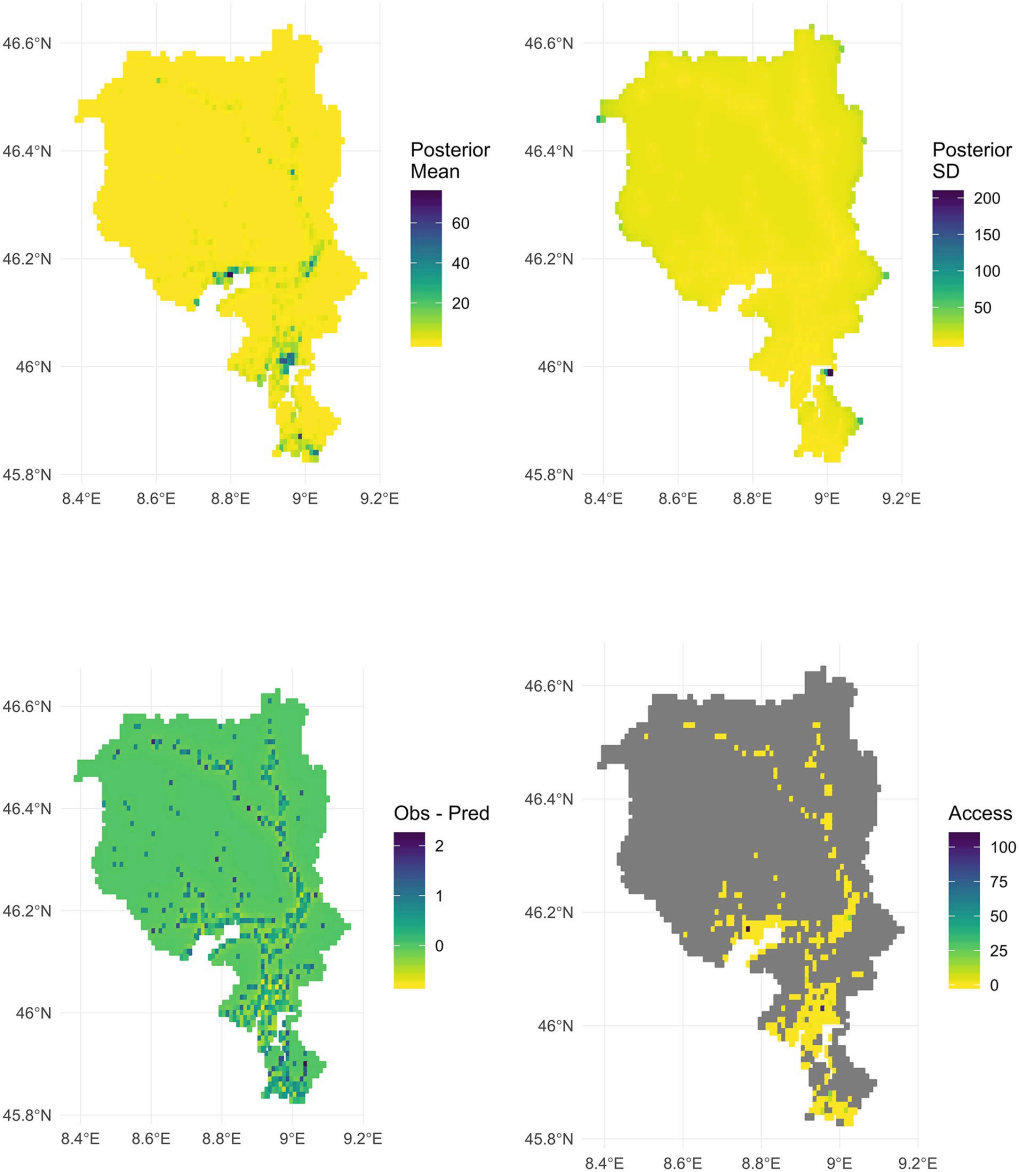
Top Left: Posterior mean estimates of the number of OHCA events in each spatial area, Top Right: Posterior Estimates of Standard Deviation of the number of OHCA events in each spatial area, Bottom Left: Error as measured by the observed—posterior mean of the number of OHCA events in each spatial area. Bottom Right: Access score areas with predicted OHCA count of less than 1 are ignored (grey).

The posterior mean, standard deviation, and residuals (observed count—posterior mean) are depicted in Fig 5. Areas with values greater than zero indicate that observed counts of OHCA are greater than model expected values. Areas with values less than zero indicate that observed counts of OHCA events are less than model expected values. Areas marked with darker colours indicate higher occurrence. The posterior mean values are similar to the observed values, and some areas have a high standard deviation at the edges of the map. The presence of possible spatial autocorrelation of the regression residuals is tested using the Moran’s I test [38, 39]. The global Moran’s I value is I = -0.03, with *p* = 0.989, indicating no significant overall spatial clustering in the residuals of the model. Areas with high difference in observed and predicted are mostly in rural areas. The top 10 grid cell locations with the most OHCA events are mostly in urban regions (Table 2). A table of the top 20 municipalities by model error is shown in Appendix B in S1 File.

The analysis reveals that almost all areas (99.1%) had an access score of less than one. Priority ranks were created and access scores of each spatial area are shown in Fig 5 (bottom right), where areas shown in grey indicate locations that have a predicted OHCA count of < 1. Tables 3 and 4 show the number of AEDs placed in the the top 20 least accessible areas. The top 10 priority areas have AEDs placed in them, but priority ranks 11 to 20 have only 5 AEDs placed, 4 of which are in rural areas. When examining the top 20 grid cell areas where AEDs are placed, the optimisation favours urban areas, which have higher model predicted OHCA counts.

**Table 3:** Top 20 priority ranks, access scores, model predictions, the number of AEDs added, and whether the areas were rural or urban.

Priority Rank	Access	Model Predicted	# AEDs Added	Urban / Rural
1	0	32.22	3	rural
2	0	23.24	1	urban
3	0	21.33	2	rural
4	0	18.77	2	rural
5	0	17.51	1	urban
6	0	14.74	1	urban
7	0	14.14	1	rural
8	0	11.10	1	rural
9	0	10.40	1	rural
10	0	10.40	1	rural
11	0	10.30	1	rural
12	0	9.57	0	urban
13	0	9.00	1	urban
14	0	8.91	1	rural
15	0	8.58	0	rural
16	0	8.31	0	rural
17	0	8.21	0	urban
18	0	8.06	1	rural
19	0	7.83	0	rural
20	0	7.79	1	rural

**Table 4:** Top 20 number of AEDs added, along with priority ranks, access scores, model predictions, and whether the areas were rural or urban.

Priority Rank	Access	Model Predicted	# AEDs Added	Urban / Rural
229	0.07	74.37	6	urban
261	0.18	43.60	5	urban
240	0.10	46.35	4	urban
1	0.00	32.22	3	rural
244	0.10	42.22	3	urban
284	0.50	44.79	3	urban
312	3.83	42.41	3	urban
3	0.00	21.33	2	rural
4	0.00	18.77	2	rural
209	0.02	24.99	2	rural
221	0.05	19.65	2	rural
223	0.06	32.32	2	rural
235	0.09	32.45	2	urban
237	0.09	19.75	2	urban
252	0.13	34.14	2	urban
255	0.15	25.86	2	rural
260	0.18	20.36	2	rural
281	0.49	33.89	2	rural
302	1.19	38.04	2	urban
2	0.00	23.24	1	urban

## Discussion

This paper presents a novel modelling framework that jointly prioritizes regions for AED placement, evaluates AED placement methods, and identifies covariates important for predicting OHCA events. This reconciles a missed opportunity in past research by [1], which did not identify exact AED locations in priority regions, and in [14], which did not combine exact AED locations with relevant spatial information important in predicting OHCA occurrence [1, 15, 16]. Combining information from geospatial models and the precise facility locations gives a more complete picture of the impact of facility locations. The geospatial model revealed an increase of OHCA occurrence for the covariates: recreational and industrial areas, and the proportion of men, proportion of the population older than 65, and the population density. Additionally, the proportion of buildings, urban areas, and financial strengths are associated with lower counts of OHCA events.

A limitation of this study is that the currently available shapefiles are at a low spatial resolution. This lead us to make the unrealistic assumption that municipalities have their covariates equally distributed within the surface area. Data at a higher spatial resolution could also help to glean more useful insight into the relationship between OHCA risk factors and AED placement. However, a main aim of this paper was to demonstrate finding factors related to OHCA occurrence, and using the floating catchment approach and AED allocation methods in evaluating priority areas and placement methodology.

The distance to an existing OHCA event is crucial to survival, but is only a measure of past occurrence. Incorporating floating catchment area models with spatial modelling of relevant spatial risk factors for OHCA events helps us understand how AED deployment strategies work in spatial areas with higher OHCA occurrence risk factors (more men, higher population density and older populations). There may be situations with scarcity of services, where multiple OHCA events happen and exhaust available AEDs. To fully account for such a situation, additional modelling would need to be made where a “wrong decision” was made, where perhaps an agent made a false trip to where an AED was located previously. These have not been modelled yet, although scenarios have been considered in [2], which modelled scenarios with many active bystanders searching for AEDs to use for treatment, and bystanders found AEDs farther away from the bystander.

Use of the binary coverage in the optimisation model means that areas are either covered or not. This means that an OHCA event 99m and 1m away from an AED location get the same weight, of 1, and an AED 101m away gets a weight of 0—it is marked as being not covered. Although accessibility score in this paper uses a exponential decay, an approach that considers continuous distance has not been considered in AED placement. Continuous distance could be incorporated, using what is known as the continuous coverage problem, described in [40, 41]. This could perhaps incorporate factors such as spatial risk or lives saved through AED placement, so that that spatial areas with higher OHCA risk could have more AEDs. Information on risk of death from an OHCA event, or perhaps some measures of accessibility, could also be included so that areas with greater risk are given more weight.

In our work and in previous literature [1], the accessibility models only used the mean posterior values from the geospatial model. The Bayesian framework can be used to recover distributions of accessibility for each spatial region, which would account for uncertainty in accessibility, and allow for calculation of probabilities of spatial regions being ranked as the lowest access, and calculation of regions in the lowest/top 10% accessibility of a region. This could be useful when determining priority rankings, allowing for questions regarding not just the access score, but the certainty of access.

In practical cases, there are many more rural areas with low access and high demand compared to urban areas, and so additional approaches are needed for servicing rural regions with low access. One response to this need is the use of drone deployed AEDs [42, 43]. These different uses of AED deployment could in the future be combined with current deployment methods, potentially included as an additional cost to regular AEDs, and also constrained, according to areas with lower access scores. Deploying AEDs and drone delivery services is an expensive endeavour, so future methods could, in addition, incorporate the use of AED deployment methods, into a relocation-type model. This could, for example, explore the use of relocating several AEDs in particular regions to one central node containing an AED delivery drone.

Alternative arrival vectors, such as those by an ambulance, may be considered for AED delivery. However, their modelling requires answering a different set of questions, concerning placement of ambulances and of ambulance stations, and accounting for additional external features such as traffic at a given time of day. Additionally, an ambulance may take up to 7 or 8 minutes to arrive on scene, which is beyond a reasonable survival time, which may mean other arrival vectors such as motorcycles may be considered.

Future research could explore different distances (100m–1km) for different modes of transport, such as healthy runners, motorcycles, and ambulances. The arrival and approach of healthy runners and an ambulance should be addressed in a different framework that assesses the full combinations of these events. This might include situations such as race conditions, where someone starts at 100m, someone runs 400m, and an ambulance arrives. This is a complex analysis, and is partially discussed in Chan et al 2016 [2], where many different optimisation formulations account for different scenarios of arrival. Furthermore, larger distances should account for available paths/routes instead of assuming a straight line of travel. Path distances are typically challenging to calculate, but modern tools such as dodgr provide accessible calculations of many-to-many pairwise distances for flow via networks, providing realistic routing through streets [44].

This paper creates priority rankings comprised of spatial risk factors and coverage information from OHCA and AED events. This information could be used to create a table of ranks and access scores that consider changes in access for different AED placements. It also provides a different perspective for evaluating AED placement methodology, allowing for different questions and decisions to be made in AED placement.

## Supporting information

S1 File Appendix A, Definition of financial strength. Appendix B, Alternative formulation of spatial regression that accounts for age and gender in an offset term. Appendix C, Top 20 Municipalities by model error count, and their model error and predictions. Appendix D, Summary of key characteristics of OHCA data. Appendix E, Stepwise and Exponential Decay. Appendix F, Figure of different access models. Appendix G, Tables of exponential and stepwise decay for priority and top AEDs.
(PDF)
