# Supplementary material for: Evaluating health facility access using Bayesian spatial models and location analysis methods
Source: PLoS One. 2019 Aug 7;14(8):e0218310. doi: 10.1371/journal.pone.0218310 (PMC6685678; doi:10.1371/journal.pone.0218310)
Supplement: S1 File — Appendix A, Definition of financial strength. Appendix B, Alternative formulation of spatial regression that accounts for age and gender in an offset term. Appendix C, Top 20 Municipalities by model error count, and their model error and predictions. Appendix D, Summary of key characteristics of OHCA data. Appendix E, Stepwise and Exponential Decay. Appendix F, Figure of different access models. Appendix G, Tables of exponential and stepwise decay for priority and top AEDs. (PDF) [file pone.0218310.s001.pdf]

Supplementary Materials for Evaluating health facility access using Bayesian spatial models and location analysis methods

Nicholas J Tierney (1,2,3), Antonietta Mira(4,5), H. Jost Reinhold (4), Giuseppe Arbia (4,6), Samuel Clifford (1,2, 7, 8), Angelo Auricchio (9,10,11), Tiziano Moccetti(9), Stefano Peluso (4,6), Kerrie L. Mengersen(1,2).

1: Department of Statistical Science, Mathematical Sciences, Science & Engineering Faculty, Queensland University of Technology, Brisbane, Queensland, Australia

2: ARC Centre of Excellence for Mathematical and Statistical Frontiers (ACEMS), Brisbane, Queensland, Australia

3: Department of Econometrics and Business Statistics, Monash University, Melbourne, Victoria, Australia

4: Data Science Center, Institute of Computational Science, Università della Svizzera italiana, Lugano, Switzerland

5: Department of Science and High Technology, Università dell'Insubria, Como, Italy

6: Department of Statistical Sciences, Università Cattolica del Sacro Cuore, Milan, Italy

7: Department of Infectious Disease Epidemiology, London School of Hygiene & Tropical Medicine, London, United Kingdom

8: Centre for Mathematical Modelling of Infectious Diseases, London School of Hygiene & Tropical Medicine, London, United Kingdom

9: Fondazione Ticino Cuore, Lugano, Switzerland;

10: Division of Cardiology, Fondazione Cardiocentro Ticino, Lugano, Switzerland;

11: Center for Computational Medicine in Cardiology, Università della Svizzera Italiana, Lugano, Switzerland

Supplementary Material A: Definition of financial strength

The definition of the Financial Strength Index (Indice di Forza Finanziaria) of the Canton Ticino municipalities can be found at: https://www3.ti.ch/DFE/DR/USTAT/allegati/comune/239agno.pdf

In Italian:

> Permette di situare la forza finanziaria di un Comune rispetto alla media di tutti i Comuni, posta uguale a 100 (riportata nel ritratto cantonale). È usato, insieme ad altri indicatori derivati, per modulare le partecipazioni dei Comuni a seconda della loro forza, ad esempio per graduare i sussidi del Cantone ai Comuni, per graduare i contributi che i Comuni versano al Cantone o per ponderare le partecipazioni finanziarie in ambito intercomunale.

In English:

> The Financial Strength Index allows comparing the financial strength of a municipality with respect to the average of all the Canton Ticino Municipalities, which is set equal to 100. It is used, along with other derived indicators, to modulate the economic participation of the municipalities according to the degree of their financial strength. For instance: it allows to graduate the subsidies of the Canton to the municipalities, and to graduate the contributions that the municipalities pay to the Canton or to weigh financial contributions between municipalities.

The law that specifies how the Financial Strength Index is computed can be found at: https://m3.ti.ch/CAN/RLeggi/public/index.php/raccolta-leggi/legge/num/71 (see art. 16)

Essentially, the Financial Strength Index for year 0 is computed as a weighted average of five indicators:

1) The per capita fiscal revenue, i.e. the ratio between the total fiscal revenue at year (-3) and the number of residents on December 31st of the year (-3). WEIGHT = 3.
2) The per capita Federal Tax revenue, per the most recently available tax year. WEIGHT = 1.
3) Percentage of taxpayers subject to the Federal Tax, i.e. the ratio between the number of taxpayers subject to the Federal Tax and the number of taxpayers subject to the Cantonal Tax. WEIGHT = 1.

4) Political Tax Multiplier. WEIGHT = 2.

5) Evolution of the Resident Population. Three rates of increase/decrease are considered: population growth between 1850 and 1950, population growth between 1950 and 1980, population growth between 1980 and the last year for which data are available. WEIGHT = 1.

Municipalities are ranked in six different categories, based in the FSI:

- Very strong: above 105
- Strong: 90.01 to 105
- Medium High: 75.01 to 90
- Medium Low: 60.01 to 75
- Weak: 45.01 to 60
- Very Weak: up to 45

Supplementary Material B: Alternative formulation of spatial regression that accounts for age and gender in an offset term

$$Y_{ki} \sim Poisson(\mu_{ki}) \tag{S2.1}$$

$$\mu_{ki} \sim E_{ki}\lambda_{ki} \tag{S2.2}$$

$$log(\lambda_{ki}) = \beta_0 + E_{ki} + u_i + v_i \tag{S2.3}$$

An offset term was calculated to adjust for age and gender effects. Here, we created the offset term for each municipality, and then divided it up uniformly across all grids. We define E_k as the expected number of events in the kth municipality, and d_j as the total number of events in group j in all of Ticino, and n_j as the total number of people in group j in all Ticino, and n_{kj} as the number of people in the age group j in municipality k:

$$E_k = \sum_{j=1}^{J} \frac{d_j}{n_j} n_{kj} \tag{S2.4}$$

We then obtain the offset for each grid within each municipality by dividing up the offset for the municipality k by the number of grids whose centroid falls within municipality, N_{gk}:

$$E_{ki} = \frac{E_k}{N_{gk}} \tag{S2.5}$$

Supplementary Material C: Top 20 Municipalities by model error count, and their model error and predictions.

Table 1: Top 20 Municipalities by model error count, and their model error and predictions.

Municipality	Model Error	Model Prediction
36	2.19	0.81
53	1.96	8.04
3	1.86	14.14
89	1.69	8.31
23	1.62	2.37
58	1.62	2.38
37	1.59	3.41
64	1.55	5.45
97	1.55	9.45
93	1.50	3.50
50	1.50	3.50
14	1.48	1.52
71	1.47	59.53
1	1.47	3.53
7	1.47	0.53
96	1.45	9.55
22	1.45	2.55
56	1.42	4.58
22	1.42	0.59
97	1.41	5.59

Supplementary Material D: Summary of key characteristics of OHCA data

Table 2: Summary of key characteristics of OHCA data

age_range	n	pct
0_14	3	0.11
15-64	742	26.48
65-100	2041	72.84
NA	16	0.57

Supplementary Material E: Stepwise and Exponential Decay

The exponential decay is given by:

$$G(d_{kj}, d_0) = \begin{cases} \frac{e^{-\frac{1}{2}\times(\frac{d_{kj}}{d_0})^2} - e^{-\frac{1}{2}}}{1-e^{-\frac{1}{2}}}, & d_{kj} \leq d_0 \\ 0 & d_{kj} > d_0 \end{cases} \tag{S5.1}$$

And the stepwise decay is given by

$$d(d_{kj}; J, m, d_0) = \begin{cases} 1 - J(\lfloor \frac{m*d_{kj}}{d_0} \rfloor + 1) & \text{if } d_{kj} < d_0 \\ 0 & \text{if } d_{kj} \geq d_0 \end{cases} \tag{S5.2}$$

where $\lfloor \cdot \rfloor$ if the floor function, J is the jump size, and m is the number of jumps before the maximal distance d_0, starting from which the decay function is null.

A plot of both the stepwise and exponential decay for different maximum distances is given below

Supplementary Material F: Figure of different access models

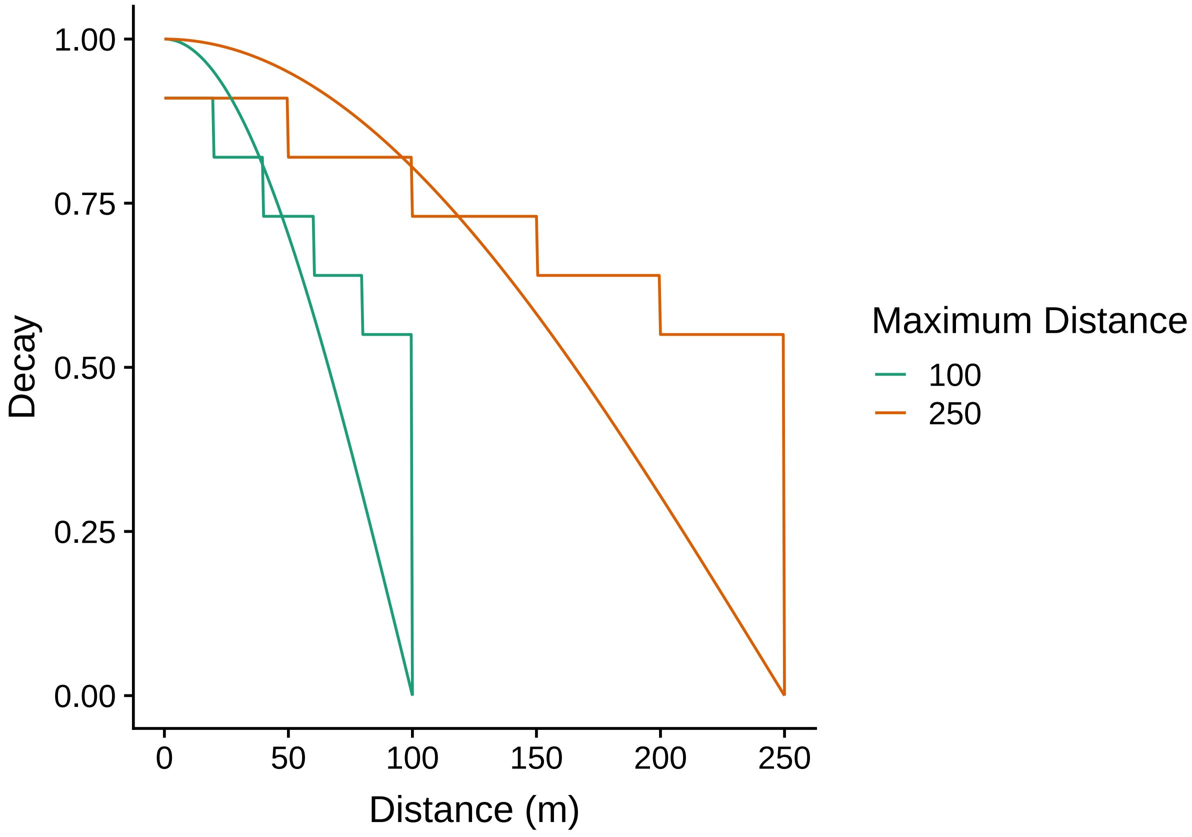

Figure 1: Exponential decay (smooth) and stewpise decay (jagged) over distances.

Supplementary Material G: Tables of exponential and stepwise decay for priority and top AEDs

These tables are replicates of Tables 3 and 4 from the main paper, but applied for the exponential decay at 250m, and stepwise decay functions at 100m and 250m. There was effectively no functional difference between the stepwise decay functions and exponential decay in terms of access score and priority ranking. Where the access models used 250m instead of 100m, the areas with low access mismatched the AED placements and predicted OHCA events. This indicates that as access stretches out to 250m, priority regions occur that are not what would be expected by the AED placement model or the OHCA predictions.

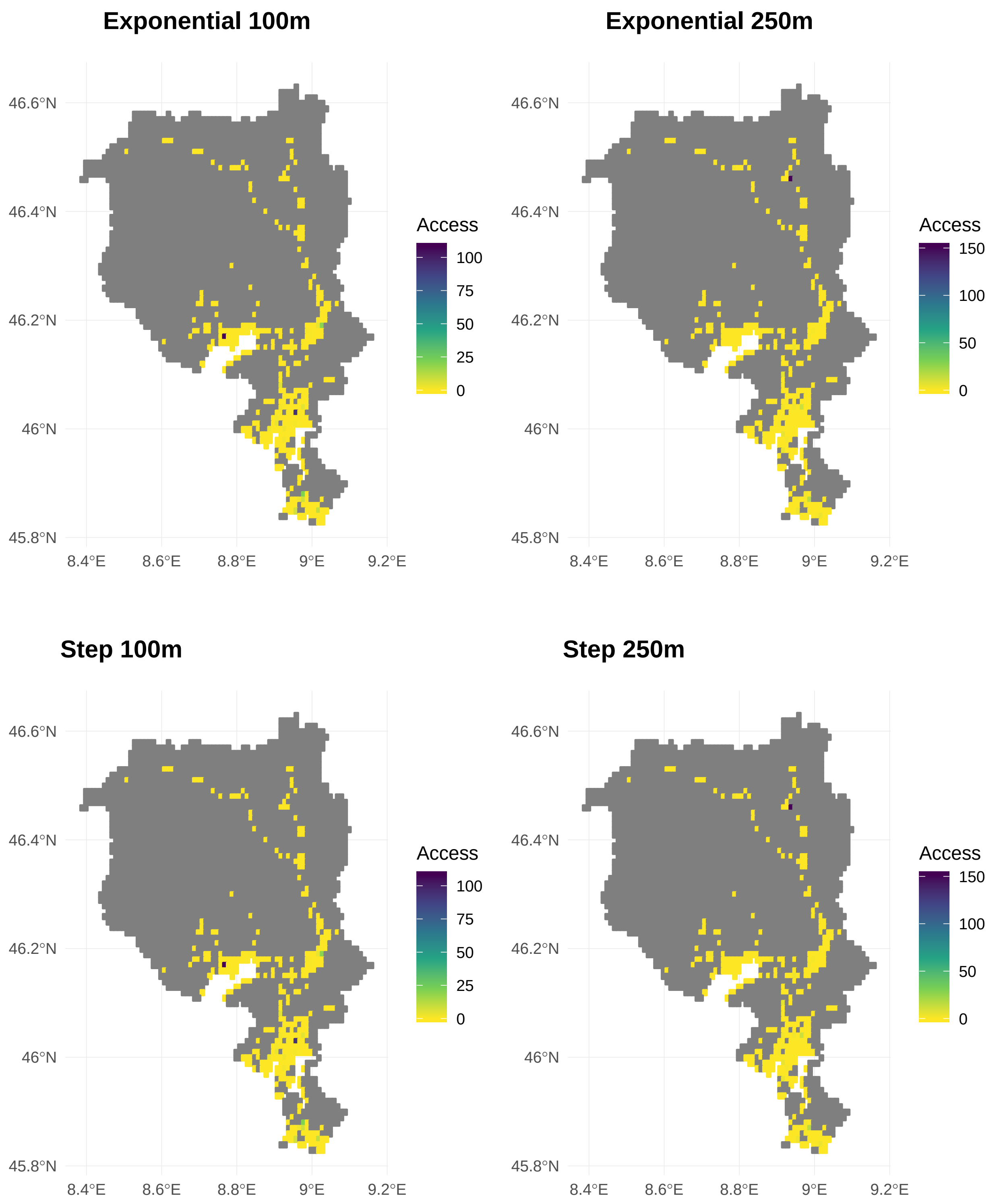

Figure 2: Map of all different access models

Table 3: Top 20 priority ranks, access scores, model predictions, the number of AEDs added, and whether the areas were rural or urban, for Exponential at 250 metres

Priority Rank	Access	Model Predicted	# AEDs Added	Urban / Rural
1	0	14.7365	1	urban
2	0	10.4019	1	rural
3	0	10.3004	1	rural
4	0	9.5683	0	urban
5	0	8.9982	1	urban
6	0	8.2136	0	urban
7	0	8.0558	1	rural
8	0	7.7851	1	rural
9	0	6.7758	1	rural
10	0	6.4183	1	rural
11	0	6.2394	1	rural
12	0	5.9908	0	rural
13	0	5.9199	1	rural
14	0	5.7307	0	rural
15	0	5.7266	1	urban
16	0	5.2685	1	rural
17	0	5.1602	1	urban
18	0	5.1189	0	urban
19	0	5.1012	1	urban
20	0	4.8331	0	rural

Table 4: Top 20 priority ranks, access scores, model predictions, the number of AEDs added, and whether the areas were rural or urban, for Step Decay at 100 metres

Priority Rank	Access	Model Predicted	# AEDs Added	Urban / Rural
1	0	32.2178	3	rural
2	0	23.2365	1	urban
3	0	21.3296	2	rural
4	0	18.7724	2	rural
5	0	17.5064	1	urban
6	0	14.7365	1	urban
7	0	14.1398	1	rural
8	0	11.0997	1	rural
9	0	10.4044	1	rural
10	0	10.4019	1	rural
11	0	10.3004	1	rural
12	0	9.5683	0	urban
13	0	8.9982	1	urban
14	0	8.9101	1	rural
15	0	8.5784	0	rural
16	0	8.3091	0	rural
17	0	8.2136	0	urban
18	0	8.0558	1	rural
19	0	7.8294	0	rural
20	0	7.7851	1	rural

Table 5: Top 20 priority ranks, access scores, model predictions, the number of AEDs added, and whether the areas were rural or urban, for Step Decay at 250 metres

Priority Rank	Access	Model Predicted	# AEDs Added	Urban / Rural
1	0	14.7365	1	urban
2	0	10.4019	1	rural
3	0	10.3004	1	rural
4	0	9.5683	0	urban
5	0	8.9982	1	urban
6	0	8.2136	0	urban
7	0	8.0558	1	rural
8	0	7.7851	1	rural
9	0	6.7758	1	rural
10	0	6.4183	1	rural
11	0	6.2394	1	rural
12	0	5.9908	0	rural
13	0	5.9199	1	rural
14	0	5.7307	0	rural
15	0	5.7266	1	urban
16	0	5.2685	1	rural
17	0	5.1602	1	urban
18	0	5.1189	0	urban
19	0	5.1012	1	urban
20	0	4.8331	0	rural

Table 6: Top 20 number of AEDs added, along with priority ranks, access scores, model predictions, and whether the areas were rural or urban, for Exponential at 250 metres

Priority Rank	Access	Model Predicted	# AEDs Added	Urban / Rural
158	0.0097	74.3663	6	urban
171	0.0239	43.5994	5	urban
197	0.0591	46.3548	4	urban
161	0.0132	32.2178	3	rural
178	0.0347	42.2157	3	urban
187	0.0414	44.7886	3	urban
246	0.2448	42.4071	3	urban
155	0.0074	24.9894	2	rural
163	0.0152	33.8931	2	rural
167	0.0192	18.7724	2	rural
177	0.0294	21.3296	2	rural
179	0.0353	32.3200	2	rural
184	0.0381	25.8574	2	rural
190	0.0430	32.4529	2	urban
196	0.0570	19.7531	2	urban
199	0.0611	19.6534	2	rural
204	0.0639	34.1371	2	urban
211	0.0852	20.3588	2	rural
239	0.1905	38.0412	2	urban
1	0.0000	14.7365	1	urban

Table 7: Top 20 number of AEDs added, along with priority ranks, access scores, model predictions, and whether the areas were rural or urban, for Step Decay at 100 metres

Priority Rank	Access	Model Predicted	# AEDs Added	Urban / Rural
225	0.0407	74.3663	6	urban
271	0.1692	43.5994	5	urban
244	0.0774	46.3548	4	urban
1	0.0000	32.2178	3	rural
242	0.0703	42.2157	3	urban
280	0.2307	44.7886	3	urban
313	0.6570	42.4071	3	urban
3	0.0000	21.3296	2	rural
4	0.0000	18.7724	2	rural
208	0.0137	24.9894	2	rural
213	0.0187	32.4529	2	urban
223	0.0371	19.6534	2	rural
227	0.0411	32.3200	2	rural
235	0.0546	19.7531	2	urban
236	0.0558	33.8931	2	rural
241	0.0673	34.1371	2	urban
243	0.0761	25.8574	2	rural
253	0.0988	20.3588	2	rural
311	0.5524	38.0412	2	urban
2	0.0000	23.2365	1	urban

Table 8: Top 20 number of AEDs added, along with priority ranks, access scores, model predictions, and whether the areas were rural or urban, for Step Decay at 250 metres

Priority Rank	Access	Model Predicted	# AEDs Added	Urban / Rural
156	0.0073	74.3663	6	urban
167	0.0154	43.5994	5	urban
188	0.0318	46.3548	4	urban
160	0.0098	32.2178	3	rural
175	0.0241	42.2157	3	urban
185	0.0287	44.7886	3	urban
240	0.1350	42.4071	3	urban
155	0.0064	24.9894	2	rural
158	0.0083	18.7724	2	rural
162	0.0116	33.8931	2	rural
165	0.0151	21.3296	2	rural
173	0.0218	32.3200	2	rural
178	0.0254	25.8574	2	rural
183	0.0270	32.4529	2	urban
202	0.0453	19.6534	2	rural
204	0.0503	19.7531	2	urban
208	0.0547	34.1371	2	urban
210	0.0566	20.3588	2	rural
237	0.1305	38.0412	2	urban
1	0.0000	14.7365	1	urban
